# Variations in Manual Dexterity in 11- and 12-Year-Old Children in the North of Spain in the SARS-CoV-2 Lockdown

**DOI:** 10.3390/ijerph19127162

**Published:** 2022-06-10

**Authors:** Oliver Ramos-Álvarez, Víctor Arufe-Giráldez, Alberto Sanmiguel-Rodríguez, Rubén Navarro-Patón

**Affiliations:** 1 Education Faculty, Elviña University Campus, University of A Coruña, 15008 A Coruña, Spain; 2 Departamento de Educación, Área de Educación Física y Deportiva, Universidad de Cantabria, Avda. Los Castros, 50, 39005 Santander, Spain; 3 Research Unit of School Sports, Physical Education and Psychomotricity (UNIDEF), Specific Didactics Department, Research and Diagnostic Methods in Education, Education Faculty, Elviña University Campus, University of A Coruña, 15008 A Coruña, Spain; v.arufe@udc.es; 4 Faculty of Language and Education, University of Camilo José Cela, 28692 Madrid, Spain; asrgz2014@gmail.com; 5 Facultad de Formación del Profesorado, Universidade de Santiago de Compostela, 27001 Lugo, Spain; ruben.navarro.paton@usc.es

**Keywords:** SARS-CoV-2, lockdown, physical activity, children, MABC-2

## Abstract

Between March and June 2019, the SARS-CoV-2 virus broke out in Spain. The lockdown in response entailed the modification of certain habits in the infant–juvenile population, such as those related to the practice of physical activity and the implications derived from it. The aim of this study was to learn the impacts that lockdown had on manual dexterity in children aged 11–12 years. Methods: A total of 50 Spanish children aged 11–12 years (M = 11.40; SD = 0.50) participated, 33 (66%) boys and 17 (34%) girls. The Movement Assessment Battery for Children 2 (Movement ABC-2) and an ad hoc questionnaire for socio-demographic data and other relevant information were administered for the data collection. Results: There are significant differences (*p* < 0.05) in the results for the manual dexterity variables measured by the peg turning and line drawing tests as well as by the measured, scalar and percentile dimension scores for manual dexterity between before and after the lockdown in both boys and girls. No significant differences were found in measurements related to the triangle assembly variable (*p* = 0.125). Conclusions: A significant negative impact of SARS-CoV-2 lockdown on manual dexterity values was evident in boys and girls aged 11–12 years.

## 1. Introduction

As a consequence of the health crisis caused by COVID-19, many countries (e.g., Italy, France, Portugal), including Spain, decided to take social measures such as home lockdowns to eradicate the transmission of the virus among the population. The Spanish government took this measure between 15 March and 21 June 2020 (98 days) under Royal Decree 463/2020 of 14 March, declaring a state of alarm for the management of the health crisis caused by COVID-19 and limiting the movement of the entire Spanish population except for essential workers [1]. Educational centres and sports schools were closed. This measure resulted in changes in habits in the child and adolescent population, especially in terms of their time dedicated to daily physical activity (PA), time spent using screen devices and eating habits [2,3,4]. In addition, these changes in habits have resulted in changes in children’s anthropometric averages, physical condition and psychological and emotional states [4,5,6].

Relying on authors such as Piaget and Gallahue, Ruiz Pérez [7,8,9,10] distinguishes different stages in the motor development of children. First is the prenatal stage, characterised by involuntary phylogenetic movements; the second stage is between birth and 2 years of age (reflex movements at first and then rudimentary movements), the stage from 2 to 6 years of age is characterised by the learning of fundamental movements (basic motor skills); the stage between 6 and 12 years of age is when the greatest motor achievements and milestones occur, some of a general nature from 7 to 10 years and others of a more specific nature between 10 and 12 years of age.

During this motor development, the use of the hands and the manipulation of objects are central to children’s learning. Poor hand control is linked to other types of difficulties, which will not only be reflected in the early years of a child’s life but may develop into problems later in life [11]. This psychomotor development is a consequence of the interaction of the child’s own biological factors with the psychosocial context. This social, affective, educational and ecological stimulation takes place through the active participation of the child in his or her motor development and thus in his or her manual dexterity. In addition, these motor experiences are fundamental for avoiding deficiencies or deterioration in motor development [12,13]. For instance, dwellings that are too small or crowded and other negative reflections of socio-economic status will limit a child’s motor and physical development [14]. 

There are different variables that can influence manual dexterity and its development. These variables are grasping, coordination and all the skills that the child has acquired through the practice of activities in which he has had to manipulate some type of object. This is why manual dexterity depends on the anatomical–physiological relationship between the central and peripheral nervous systems and external qualities such as learning, training and the experience of the person performing the action. Different studies have shown that movement for a given purpose depends on the person’s knowledge of the occupation of his body in space, where he intends to direct the movement of his body and the choice of a motor plan to achieve the proposed objective [15,16].

Taking into account these psychosocial conditions, children’s manual dexterity and fine motor skills should be consolidated by the age of about 9 years [17], and educational stimulation, particularly in physical education classes, can be a favourable context for working on and improving fine motor skills [18]. 

The school stage is characterised by a motor development in which motor–perceptual capacities acquire a certain balance thanks to the maturation of the central nervous system and motor learning, the acquisition of the body schema and a greater functional autonomy of the body segments [7]. Much of the maintenance of this balance is due to exogenous factors such as the practice of PA [19]. However, deficits in physical fitness and its most direct consequence, poor physical condition, are related to motor development problems [20,21], among which are low levels of manual dexterity. By the age of 11–12 years, all coordination skills should be fully defined and functionally efficient [7].

It should be noted that regular PA becomes a fundamental element in this motor development. For the proper development of children and adolescents, the World Health Organization (WHO) published among its PA practice recommendations for the world population that school-age children should engage in at least 60 min of moderate to vigorous intensity PA (MVPA) daily. Furthermore, the WHO stated that if PA practice exceeds 60 min per day in children, its health benefits will increase [22]. Likewise, this institution affirms that children between 5 and 12 years of age should use technological devices and screens for no more than 60 to 90 min a day [22,23]. 

In addition, regular PA at a minimal intensity of exertion, as well as having health benefits, can prevent infectious diseases. Practising regular PA at least 30 min a day, 5 days a week can reduce the risk of contracting a virus, including COVID-19, by 31%, reduce the likelihood of death from infectious diseases by 37% and improve the effectiveness of vaccines by up to 40% [24].

The recommendations by the WHO are far removed from the realities of Spanish children aged between 9 and 15 years. The highest levels of sedentary lifestyles and abandonment of PA and sports outside the school context are found in these ages, with 85% of girls and 78% of boys not performing the minimum minutes established by the WHO [23]. Also of concern is the population of children who seem to be most in need of regular PA practice, such as schoolchildren who are overweight or obese compared with schoolchildren with normal-weight body mass index (BMI) [25]. 

In addition to this abandonment of PA and sports practice at school age, there is another important factor that does not comply with the WHO’s global recommendations: the time spent using technological devices and viewing screens. Taking as a reference the data provided by this institution, the time spent using technological devices and the screen viewing in the school population is much higher than the WHO recommendations [26]. Both elements, the decrease in the practice of PA and sports and the increase in the time spent using technological devices and screens, constitute the so-called technological sedentary lifestyle [2,27] and may be associated with other health problems [28].

Thus, the main objective of this research is to find out whether there has been an impact on manual dexterity in 11- and 12-year-old children as a consequence of the SARS-CoV-2 lockdown in a region of northern Spain. We investigated the possible relationships established between the assessed manual dexterity tests and certain socio-demographic variables (e.g., type of housing, place of residence, time spent in PA) in the context of the closure of schools.

## 2. Materials and Methods

### 2.1. Study Design

A descriptive and longitudinal observational study was carried out [29]. Children’s manual dexterity was used as the dependent variable. In order to define the independent variables of the research, an ad hoc questionnaire was used to collect socio-demographic data (e.g., age, sex, parents’ level of education, employment status) on the participants in the study as well as data concerning the different variables under study (e.g., perception of tiredness, perception of self-esteem, perception of creativity).

### 2.2. Participants

A total of 55 children were invited to participate in the research from a primary school located in the north of Spain who were enrolled in the sixth grade. The school was located in a semi-urban residential area close to the city of Santander. A total of 50 children ultimately participated in the study, 33 (66%) boys and 17 (34%) girls (median age = 11.40; SD = 0.50) as the other 5 were excluded from the study because they did not present the informed consent from their parents or legal guardians or because they decided not to participate. Of the total sample, 56% resided in an urban setting, and 38% resided in a semi-urban or residential setting; only 6% resided in a rural setting during the period of the SARS-CoV-2 lockdown.

### 2.3. Tools

The Movement Assessment Battery for Children 2 (MABC-2) [30], in particular its adaptation in Spanish [31], was the instrument used to collect the data for the research. This battery is made up of three dimensions: Dimension 1, which assesses the children’s manual dexterity, Dimension 2, which assesses aiming and catching and Dimension 3, which assesses balance. 

Each of these dimensions is made up of different tests. For this research, we used the results obtained from the Dimension 1 tests of manual dexterity as well as their reference values established in the MABC-2 [31]. The tests administered in this research were the turning of pegs with the dominant and then the non-dominant hand, the assembly of a triangle in the shortest possible time and the drawing of a line. 

In the turning of pegs, the child must reverse the pegs on the board with only one hand, first the dominant hand and then the non-dominant hand. The pegs must be placed in the same hole, and the test is timed until the child turns the last peg. The child has two trials and is considered null or unsuccessful if after two attempts he/she does not manage to complete the test (dropping the peg, touching a peg with the other hand or another part of the body). The triangle assembly test consists of assembling a triangle with bars, bolts and nuts according to a model. The test is timed and includes a pre-test and two attempts to complete the test in the shortest possible time. The test is considered a failure if the assembly is incorrect, if the material the respondent has already picked up is put back on the table, if the respondents lean the material on their bodies or if they drop material out of their reach. Finally, the test of drawing with a continuous line (respondents may not lift the pen, go back or change the angle of the paper by more than 45°) requires following a set path between two lines. The child has a rehearsal before the test and two attempts to perform the test with his/her dominant hand. The test is considered a failure if the child changes the direction of the line or turns the angle of the paper more than 45º. The test is evaluated according to the established errors: crossing the limits of the route, going outside the two limits by more than 12 mm, discontinuous strokes or blank spaces or overlapping or double strokes.

For the execution of the tests, their measurement and their subsequent evaluation, the procedures established in the MABC-2 were followed. For this purpose, the specific test materials were used, as well as a digital hand-held stopwatch and a biro. 

In addition to the results obtained with the MABC-2, parents or legal guardians filled in a socio-demographic questionnaire. This questionnaire collected information on different variables: economic and education information on the family, information on the time dedicated to the practice of PA, information on the time dedicated to as well as the type of sedentary activities carried out and finally information on the time dedicated to and the type of technologies used by the children before and during the seclusion. To obtain this information, an ad hoc questionnaire was designed consisting of 50 dichotomous items, Likert-scale rated items and open-ended questions. The questionnaire showed an acceptable Cronbach’s alpha coefficient (α = 0.71) [32,33].

### 2.4. Procedure

Data collection took place during the 2019–2020 academic year. The first two data collections took place in physical education classes during the weeks of 14 October 2019 and 2 March 2020. From 15 March 2020, a period began in which all schools in Spain were closed indefinitely [1]: That was the date on which the national closure began in Spain as a result of the state of alarm decreed due to the health emergency caused by SARS-CoV-2. In order to continue with this research, all the families of the participating children were contacted and informed of the changes to the research that would be in effect for the third data collection.

This new information, transmitted in writing, did not cause any participant to drop out of the study. The research continued with the third and final data collection immediately after the end of the homebound period and the beginning of the de-escalation period on 28 May 2020. The same procedures for the instruments used in the previous data collections were used. However, modifications were made to the data collection process to ensure compliance with the health measures established by the Spanish government to prevent the spread of SARS-CoV-2. This new process, outside of physical education classes, was carried out by convening groups of six children from the sample in different time slots and in an outdoor space. With this third data collection, a statistical analysis was performed between the results obtained between pre-lockdown 2 and post-lockdown 2.

With the end of the lockdown period at home and with continuing data collection, data and information were also obtained through the socio-demographic questionnaire, a paper survey completed by parents or legal guardians.

### 2.5. Statistical Analysis 

The statistical software SPSS v. 26 (IBM Corporation, New York, NY, USA) was selected to carry out the statistical analyses of the research. For this statistical analysis, descriptive analyses of the main research variables were carried out, as well as normality tests of the quantitative variables for the testing of the hypotheses. The Kolmogorov–Smirnov statistic (*n* > 50) was used for the normality analyses of the total sample. In the case of normality tests by sex, the Shapiro-Wilk statistic (*n* < 50) was used. 

Finally, for the paired-samples test, the Mann-Whitney U test was used for the total and results by gender. This test was used to check for statistically significant differences (*p* < 0.05) between the data obtained in the different collections for the research variables. The test was performed on the two pre-lockdown data collections, as well as on the pre-lockdown 2 data and the post-lockdown data. This decision was based on having a sample of fewer than 25 female participants. 

In the case of three or more groups, the Kruskal-Wallis H-test was used. This was the case with the data collected from the family habits questionnaire.

### 2.6. Ethical Aspects 

The ethical and deontological principles established by the American Psychological Association have been followed for this research [34], as well as ethical recommendations for educational research [35]. 

Approval of the research protocol was requested from EDUCA’s Ethics Committee, which granted approval under code 82019.

## 3. Results

### 3.1. Manual Dexterity Assessment Tests and Scores

#### 3.1.1. Descriptive and Functional Analysis

The Shapiro-Wilk test for normality in assessment scores and pre-lockdown manual dexterity tests rejects the hypothesis of normality in girls. The findings for boys also reject the hypothesis of normality except for the tests of drawing a trace (*p* = 0.003), catching the ball with the dominant hand (*p* = 0.001) and catching the ball with the non-dominant hand (*p* = 0.001). In the case of the results for the post-lockdown normality test, the findings for the boys reject the hypothesis of normality except in the drawing of the trace (*p* = 0.001). In girls, the hypothesis of normality is not supported except for on the triangle assembly (*p* = 0.000) and line drawing (*p* = 0.001) tests. We tested for significant differences in the total manual dexterity score, scalar score and percentile (Table 1). 

Between the first and second pre-lockdown data collections, there are statistically significant differences in all assessment scores and manual dexterity tests performed, both in the total sample and by gender. With the exception of the line drawing test, there were no statistically significant differences in the total sample (*p* = 0.196), for the boys alone (*p* = 0.086) or for the girls (*p* = 0.698). Therefore, there was an improvement in the results for the three manual dexterity tests between the two pre-lockdown data collections, although some were results were not significantly different for boys (*p* = 0.086) or girls (*p* = 0.698).

Between pre-lockdown 2 and post-lockdown, the results again showed statistically significant differences (*p* < 0.05) in the means for all manual dexterity assessment test scores in the sample except for the triangle assembly, as well as in post-lockdown scores.

In this study, a clear worsening of the test results was evident. In the total sample, significant differences were observed in the scores on all the assessment and manual dexterity tests performed with the exception of the triangle assembly test (*p* = 0.125). The results obtained by gender showed significant differences in all assessment and manual dexterity test scores in boys. In girls, significant results were obtained on the peg turning test, with both the dominant (*p* = 0.019) and non-dominant (*p* = 0.006) hands, and on the drawing the line test (*p* = 0.012). The rest of the results obtained, despite not being statistically significant, also show a worsening. These worse results were similar to the results from the first data collection or even worse, although one exception should be noted. This exception occurs in the mean result for the sample on the triangle assembly test in that the results are much lower than the initial scores of the research. Specifically, the students’ time spent on test execution increased by more than 250.77% in the boys and 596.68% in the girls.

#### 3.1.2. Evolution of the Tests

The results of the manual dexterity of the MABC-2 assessed had significant improvements between pre-lockdown 1 and pre-lockdown 2. This did not happen in the results obtained between pre-lockdown 2 and post-lockdown, with worsening via a return to the initial values of the research (pre-lockdown 1 values). This worsening occurs in the mean values of the sample as well as by gender.

**Figure 1:**
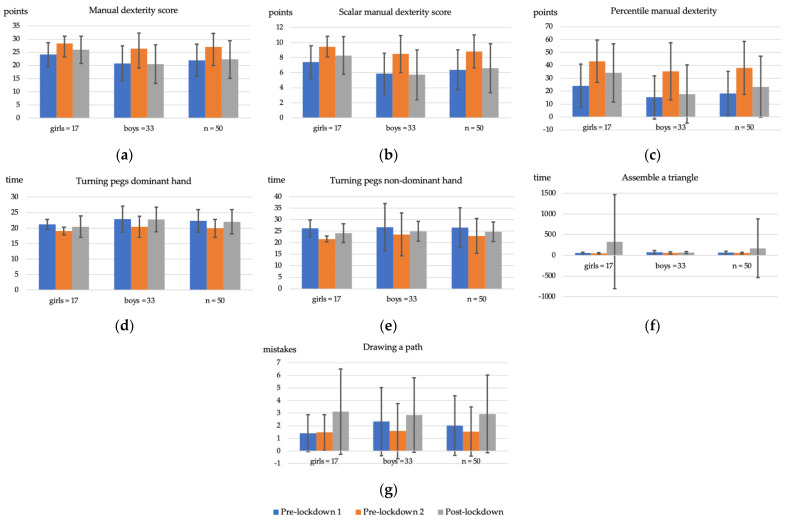
Columns of the evolution of mean manual dexterity scores and their different tests with standard deviation bars. (**a**) Manual dexterity dimension score. (**b**) Manual dexterity scalar dimension score. (**c**) Manual dexterity percentile. (**d**) Flipping pegs preferred hand. (**e**) Flipping pegs non-preferred hand. (**f**) Setting up a triangle. (**g**) Drawing the layout.

The evolution of the results obtained pre-lockdown 1, pre-lockdown 2 and post-lockdown on all the manual dexterity assessment tests as well as of the different scores on Dimension 1 of the MABC-2 is shown below (Table 1 and Figure 1). These results are broken down by the mean value for the total sample, as well as by gender, in the tests performed as well as in the total, scalar and percentile scores of manual dexterity. 

If we take into account the benchmarks presented in the MABC-2 [31], three ratings are established based on the total scores: specifically, a red zone if the total score is below 62 points (movement difficulty), an amber zone if the score is between 63 and 69 points (at risk of having movement problems) and a green zone for a score more than 69 points (no movement problems detected). The baseline mean values from the first data collection were found to be in the green zone for the full sample (M = 71.220), for the boys (M = 70.636) and for the girls (M = 72.352).

In the second data collection, still pre-lockdown, the sample continued to be in the green zone, but with markedly improved results. The mean values improved by 19.23% for the sample (M = 84.920), by 19.60% (M = 84.484) for the boys and by 18.53% for the girls (M = 85.764).

However, the latter results were drastically influenced by the period of lockdown due to SARS-CoV-2 in Spain, with a notable worsening. This was reflected in the fact that the means for the boys (M = 62.636) and for the full sample (M = 65.580) obtained results that placed them in the amber zone, i.e., the full sample but specifically the boys may be at risk of having movement problems. In the case of the girls, they remained in the green zone but were also worse than the initial values (M = 71.294).

The same evolution occurs in the specific manual dexterity test scores. Taking into account the scores on the different tests, specifically the scalar scores, it can be seen that the sample starts from low levels of manual dexterity. According to the scales established by the MABC-2, the mean scalar scores of the sample (M = 6.40) are very close to being at risk of having manual dexterity problems (≤6). In the case of the girls’ manual dexterity scalar score, the values are at the mean (M = 7.41), but the boys’ manual dexterity movement risk scores (M = 5.87) are below it.

Immediately prior to lockdown, the mean manual dexterity scalar scores were as follows: in the full sample (*p* = 8.82), in boys (M = 8.48) and in girls (M = 9.47).

However, the impact of lockdown due to SARS-CoV-2 also shows a worsening of manual dexterity in the sample, with the boys’ manual dexterity scores falling within the risk of problems (M = 5.72). The mean for the sample suffers a decrement until it is almost in the risk zone (M = 6.60); girls also suffer a worsening of their manual dexterity results (M = 8.29), but it is less shocking than in the case of boys.

Both in pre-lockdown 1 and post-confinement, the results show that the sample started the study with low manual dexterity values. They also show that despite the improvement achieved at the second data collection, there was a significant decrease in values as a consequence of the period of lockdown.

### 3.2. Family Habits

#### 3.2.1. Descriptive Analysis

A basic descriptive analysis of the data obtained by the questionnaire related to family habits during the period of lockdown was carried out. The main findings of the questionnaire showed that during the period of lockdown:

1. Exposure to screens: There was daily screen exposure of more than 60 min, with 52% using a game console, 50% watching television, 48% using the computer, 30% using a tablet and 26% using a mobile phone.
2. Educational and/or cultural activities: 82% of the sample spent more than 60 min doing homework, while 30% spent more than 146 min. Regarding other activities, 38% spent between 16 and 30 min a day reading, 2% played musical instruments and 8% spent more than 60 min on artistic activities.
3. Rest time measured in hours of sleep: 20% of the sample slept 8 h a day, 40% slept for 9 h and 38% slept for 10 h a day.
4. PA practice: There was an increase from 4% to 32% of the children in the sample who were not doing any PA. During the lockdown, the children displayed the following decreases in the frequency of weekly PA practice during lockdown: 10% decrease in practice from 2 to 3 times per week, 14% between 4 and 5 days and from 10% to 6% in children who had practiced between 6 to 7 days.
5. Place and size of residence during lockdown: Both the environment and the space available to carry out PA during lockdown were considered important information in this research, as they could be conditioning factors for the practice of PA and its repercussions for the manual dexterity of the children in the sample. Of the sample of young people, 6% resided in a rural environment, 38% in a semi-urban or residential environment and 56% in an urban environment.

Regarding the size of the dwelling, 36% of the sample resided in a house with a garden, 28% resided in a flat between 91 and 120 m^2^, 28% resided in a flat between 61 and 90 m^2^ and 8% of the sample resided in a flat of less than 60 m^2^. 

Both the place of residence and the size of the dwelling had no significant influence on the differences in the minutes of PA or on the manual dexterity scores during lockdown.

#### 3.2.2. Statistically Significant Differences

The tests used to measure whether there were statistically significant differences between the manual dexterity variables and the socio-demographic variables at pre-lockdown 2 and post-lockdown were the Mann-Whitney U test and the Kruskal-Wallis H test, taking as a reference the recommendations established by the WHO [36]. According to the items of interest for this research, these tests of independence did not show statistically significant differences (*p* > 0.05) between the post-lockdown manual dexterity variables and the socio-demographic variables, with some exceptions. 

Among these exceptions, the results that showed statistically significant differences are: the mean number of minutes/day that the child uses a video game console with respect to the triangle assembly test (*p* = 0.016) and the turning of pegs with the dominant hand with respect to the minutes/day dedicated to reading (*p* = 0.016). 

#### 3.2.3. Post-Lockdown Results

Once the analyses of the family habits questionnaire and the post-lockdown manual dexterity score and test variables had been carried out, an analysis was performed between both types of variables. 

Table 2 shows the main findings in the results for the post-lockdown manual dexterity variables in relation to the frequency of PA practice. The results show notable worsening among children who did not practice PA: increases of 37.68% and 7.32% in the time spent turning pegs with the dominant and non-dominant hands, respectively. A 636.90% increase in the time spent on triangle assembly and a 200% increase in the number of errors made in the drawing of a line were also observed. However, the children in the sample who practised PA also showed a worsening on certain tests, although in smaller percentages: a worsening of 10.10% in the children who practised PA 2–3 times a week and 14.06% in the children who practised PA 4–5 times a week on the test of drawing a path. The children in the sample who practised PA 6–7 times a week had an increase of 14.25% in time to complete the dominant hand peg-turning test and of 18.12% on the triangle assembly test. 

Table 3 shows the results obtained for the variables analysed using the manual dexterity tests in relation to the time the sample spent using screens during the confinement period to the detriment of PA practice. The results show that more time spent using technological devices did not lead to worse results on the manual dexterity assessment tests. Some tests even showed better results, specifically more minutes of use of technological devices. 

Similar results were found in the analysis of the time the sample spent on other sedentary activities during the period of lockdown such as homework, reading, playing musical instruments and artistic activities. The results showed that manual dexterity values did not worsen in relation to the daily minutes spent in these activities. 

## 4. Discussion

The aim of this study was to determine the impacts that lockdown in Spain due to the outbreak of the SARS-CoV-2 virus had on manual dexterity in schoolchildren aged 11–12 years.

The results of this study show a negative evolution in the manual dexterity scores of a sample of 11- and 12-year-old boys and girls living in a region in the north of Spain as a consequence of the lockdown due to SARS-CoV-2. This evolution is manifested in an increase in the execution time for the tests performed and an increase in the number of errors. On the first of the tests performed, the dominant and non-dominant hand peg turning, the execution times increased by 10.48% and 7.77%, respectively. On the triangle assembly test, the execution time increased by 203.20%, and the number of errors in drawing the given layout increased by 90.90%. The main consequence of the lockdown according to the post-lockdown values was decreases in the scores on the evaluated skill dimensions: the total score of manual dexterity (−15.61%), the scalar score (−22.16%) and the percentile of manual dexterity (−38.56%).

According to the reference scales and classifications presented in the MABC-2 [31] for total test scores, the sample, in addition to a worsening of the manual dexterity scores, had worse scores for the total evaluations of their movements. Pre-lockdown, the entire sample was in the so-called green zone (over 69 points). However, post-lockdown, the mean values for the full sample (*p* = 65.580) and forthe boys (*p* = 62.636) were in the amber zone (between 63 and 69 points), which may indicate a risk of movement problems. Although the girls in the sample remained in the green zone, their post-lockdown scores were significantly worse than their scores pre-lockdown, *p* = 85.764 and *p* = 71.294, respectively.

The results have provided evidence that there were changes in the measured scores for manual dexterity in the sample between pre-lockdown 2 and post-lockdown. These changes between pre-lockdown 2 and post-lockdown were significant for all of the boys’ scores. Likewise, the mean values for the total sample were significant except for the triangle assembly test. In the case of the girls, the scores with significant differences were for the peg turning test with both the dominant and the non-dominant hand as well as on the line drawing test. The results show that the time taken to execute the tests as well as the number of errors in the execution of the tests increased significantly between pre-lockdown 2 and post-lockdown. The main consequence of this was a worsening in the scores on the skill dimensions evaluated: the total manual dexterity score, the scalar score and the manual dexterity percentile. 

The results of this research confirm the scientific evidence that different studies have shown of differences in manual dexterity performance between boys and girls. These studies show that girls have better results for global motor coordination reflected in their better performance on fine motor tasks. These same studies confirm that boys perform better than girls on tasks related to throwing and aiming [37,38].

This study has also confirmed that the sample studied modified certain of its habits between pre-lockdown and post-lockdown and that these changes had an influence on manual dexterity. This change in habits has been evinced in different studies and with direct implications in different spheres of the population [2,39,40]. The most significant modification of these habits was the increases in the time of exposure to technologies and in screen time. That is, there were increases in the time spent using technology and in the time spent on sedentary activities, and there were notable decreases in the daily time spent doing PA and in the number of days per week spent doing PA to less than the WHO’s worldwide recommendations [23]. This aspect, which was exacerbated during the current health crisis due to SARS-CoV-2, has led some governments to implement measures to limit the use of technology, prevent addictions and mitigate the high levels of sedentary lifestyles among children and adolescents [41].

However, despite the negative consequences of the excessive use of new technologies to the detriment of physical activity, there are studies that show positive aspects of their use. Some studies show that the use of new technologies can favour development and promote better manual dexterity values. These studies have shown that the use of video game consoles can lead to an improvement in manual dexterity as well as to improvements in visual-motor coordination, visual-spatial capacity, hand-eye coordination, speed of information processing and decision-making [42,43].

Similarly, it has been shown that touchscreen devices such as smartphones and tablets can lead to improvements in children’s fine motor skills. A UK study of children aged 6–36 months found that children who interacted with touchscreen devices early on performed better on fine motor skills tests [44].

Finally, in reference to the changes in habits shown in this study and their direct impacts on the physical fitness of this child population including their performance on manual dexterity tests [4], it is with regard to rest and recuperation where the greatest changes are demonstrated. In the aforementioned recommendations published by the WHO, children aged 11–12 years should sleep at least 11 h a day. None of the children in this study sample complied with this recommendation [4].

In recent years, it has become evident that the child and adolescent population is far from complying with the recommendations made by institutions such as the WHO for the practice of PA. The WHO states that this segment of the population does not comply with the recommended time for them to lead an active and healthy life [36,45]. This fact has been accentuated in Spain as a consequence of the great lockdown of COVID-19, worsening the physical conditioning and anthropometric parameters of children and adolescents [4,5,45,46]. Manual dexterity is closely related to fitness levels [7,19], so this worsening may be one of the causes of the decrease in their manual dexterity values. 

In these circumstances, the scientific evidence [24,47,48] confirms that optimal physical condition scores and regular PA practice are preventive tools against COVID-19 infection as well as for reducing suffering from the disease with lower health risks in case of infection. This evidence is supported by the anti-inflammatory, anti-fibrotic and antioxidant effects of regular PA practice, which can mitigate the negative effects that COVID-19 can have on the body.

Like any other research, this study has certain limitations, such as the sample size. This limitation means that the results obtained cannot be generalised to the whole population. 

Despite this, the different studies cited in this article show similar profiles to the characteristics of the sample presented in this study. The same circumstances are evident in other types of documents consulted, such as governmental and institutional documents. An example of these documents consulted is the latest survey by the Spanish National Statistics Institute (INE) in 2020 [49] and the EUROSTAT reports [50]. These documents describe similar population characteristics in terms of PA practice and eating habits to the sample studied in this research. 

## 5. Conclusions

This decline in manual dexterity values may have a multifactorial origin [31]. The factors that may have influenced this worsening of manual dexterity are the decrease in PA practice and the increase in time spent in sedentary activities. If we focus on the analysis results by gender, a more pronounced worsening in the scores for manual dexterity is evident in boys compared with girls. This difference is evident in the fact that the boys’ post-lockdown manual dexterity scores are in the amber zone. However, it should be noted that one of the limitations of the research is the difference in the sample size by gender. 

Bearing in mind that it is not possible to generalise the results of this research to the general population of this age group, this research shows the need to strengthen and work on more effective strategies to promote the practice of healthy and regular PA among children and adolescents. For this reason, work should continue to promote and develop effective strategies for adherence to the practice of PA among the children participating in this research. Only by working on this adherence, including through promoting new forms of PA practice in the home, will we be able to ensure that physical condition and movement assessment findings will not be even worse in the event of a new home confinement as described in this research.

Similarly, the children participating in this research should be provided with effective strategies for the responsible use of new technologies, especially in situations such as the large-scale lockdown due to SARS-CoV-2. In order to implement these strategies, it is recommended that they be carried out in a collaborative manner between the main agents of the socialisation of children and adolescents: families, educational centres and sports organisations, fundamentally.

In short, it is becoming increasingly urgent to establish a strategic health plan in the educational centres of research related to the practice of PA. The low levels of PA among children and adolescents, the high rates of child overweight and obesity and the very harmful consequences that the great confinement in Spain has had on the sample are more than sufficient reasons to focus efforts on working on these strategies. 

## Figures and Tables

**Table 1:** The manual dexterity test results between pre-lockdown 1, pre-lockdown 2 and post-lockdown using descriptive analysis.

	Pre-Lockdown 1			Pre-Lockdown 2			Post-Lockdown		
	*n* (Total)	Boys	Girls	*n* (Total)	Boys	Girls	*n* (Total)	Boys	Girls
SD	21.92 ± 6.16 *	20.78 ± 6.64 *	24.11 ± 4.52 *	27.12 ± 5.06	26.45 ± 5.85	28.41 ± 2.71	22.32 ± 7.16 ***	20.45 ± 7.38 ***	25.94 ± 5.17
SDS	6.40 ± 2.63 *	5.87 ± 2.73 *	7.41 ± 2.15 *	8.82 ± 2.19	8.48 ± 2.46	9.47 ± 1.37	6.60 ± 3.26 ***	5.72 ± 3.30 ***	8.29 ± 2.49
MDP	18.34 ± 17.13 *	15.29 ± 16.73 *	24.08 ± 16.88 *	38.04 ± 20.66	35.36 ± 22.31	43.23 ± 16.40	23.37 ± 23.71 ***	17.78 ± 22.57 ***	34.23 ± 22.65
SM1.1	22.34 ± 3.60 *	22.93 ± 4.20 *	21.19 ± 1.56 *	19.94 ± 2.91	20.38 ± 3.40	19.08 ± 1.27	22.03 ± 3.93 ***	22.83 ± 3.97 ***	20.47 ± 3.43 ^+^
SM1.2	26.50 ± 8.57 *	26.65 ± 10.28 *	26.23 ± 3.64 *	22.88 ± 7.61	23.54 ± 9.30	21.60 ± 1.33 **	24.66 ± 4.23 ***	24.93 ± 4.36 ^+^	24.15 ± 4.05 ^+^
SM2	67.14 ± 35.19 *	73.57 ± 39.78 *	55.22 ± 20.90 *	55.53 ± 20.64	58.22 ± 23.28	50.31 ± 13.34	168.37 ± 709.15	65.26 ± 25.05 ^+^	329.49 ± 1137.82
SM3	2.02 ± 2.37	2.33 ± 2.70	1.41 ± 1.46	1.54 ± 1.95 **	1.57 ± 2.19	1.47 ± 1.41	2.94 ± 3.08 ***	2.84 ± 2.95 ^+^	3.11 ± 3.40 ^+^

Note. Data are presented as mean ± standard deviation. *n* = 50; boys = 33; girls = 17. SD: score dimension manual dexterity; SDS: score dimension scaling manual dexterity; MDP: manual dexterity percentile; SM1.1: skills manual 1.1—flipping pegs with preferred hand; SM1.2: skill manual 1.2—flipping pegs with non-preferred hand; SM2: skill manual 2—assembling a triangle; SM3: skill manual 3—drawing the layout. * *p* < 0.001 = significant for pre-lockdown 2. ** *p* < 0.05 = significant pre-lockdown 1. *** *p* < 0.001 = significant with pre-lockdown 2. ^+^ *p* < 0.05 = significant with pre-lockdown 2.

**Table 2:** Pre- and post-lockdown manual dexterity test scores in relation to the frequency of children who were not physically active using descriptive analysis.

	PA Practice			
	No PA	2–3 Times/Week	4–5 Times/Week	6–7 Times/Week
SM1.1 pre-lockdown	19.69 ± 1.12	22.54 ± 3.64	22.00 ± 4.50	20.70 ± 3.64
SM1.1 post-lockdown	22.98 ± 4.32	21.20 ± 3.13	21.63 ± 4.70	23.65 ± 2.10
SM1.2 pre-lockdown	23.22 ± 0.24	24.35 ± 3.53	25.09 ± 5.44	25.11 ± 2.74
SM1.2 post-lockdown	24.92 ± 3.25	24.61 ± 3.75	24.65 ± 5.65	23.16 ± 7.96
SM2 pre-lockdown	52.48 ± 8.32	55.59 ± 17.47	325.96 ± 1100.65	62.40 ± 41.06
SM2 post-lockdown	386.73 ± 1211.69	49.29 ± 20.58	20.58 ± 57.90	76.21 ± 51.02
SM3 pre-lockdown	1.00 ± 0.00	2.87 ± 3.07	3.20 ± 3.36	3.00 ± 2.91
SM3 post-lockdown	3.00 ± 2.12	3.16 ± 3.65	3.65 ± 3.00	1.00 ± 1.00

Note. Data are presented as mean ± standard deviation. PA: physical activity; SM1.1: skills manual 1.1—flipping pegs preferred hand; SM1.2: skill manual 1.2—flipping pegs non-preferred hand; SM2: skill manual 2—assembling a triangle; SM3: skill manual 3—drawing the layout.

**Table 3:** The post-lockdown manual dexterity test results according to exposure to screens during lockdown using descriptive analysis.

	Average Minutes/Day of Video Game Console Use during Lockdown								
Test	0	1–15	16–30	46–60	61–75	76–100	101–115	116–130	+146
SM1.1	22.49 ± 4.77	24.63 ± 0.00	23.26 ± 3.13	22.37 ± 2.89	-	20.16 ± 2.32	-	21.06 ± 2.97	24.36 ± 6.02
SM1.2	26.35 ± 5.33	27.82 ± 0.00	24.40 ± 2.89	22.26 ± 3.39	-	21.58 ± 2.35	-	24.41 ± 3.68	25.01 ± 2.73
SM2	44.76 ± 12.85	113.00 ± 0.00	34.25 ± 0.00	86.75 ± 33.52	-	820.82 ± 1849.58	-	5814 ± 19.79	53.25 ± 8.87
SM3	2.75 ± 3.49	7.00 ± 0.00	2.00 ± 1.41	5.80 ± 5.31	-	2.66 ± 2.80	-	2.60 ± 1.95	1.60 ± 1.14
	**Average Minutes/Day of Television Use during Lockdown**								
**Test**	**0**	**1–15**	**16–30**	**46–60**	**61–75**	**76–100**	**101–115**	**116–130**	**+146**
SM1.1	26.40 ± 7.16	21.01 ± 2.63	20.94 ± 3.28	21.66 ± 1.99	-	20.44 ± 2.17	15.75 ± 0.00	21.99 ± 3.45	22.84 ± 6.80
SM1.2	27.25 ± 4.25	26.22 ± 2.97	21.93 ± 1.37	24.35 ± 3.93	-	21.94 ± 2.06	17.81 ± 0.00	24.27 ± 3.62	26.66 ± 7.31
SM2	47.18 ± 15.96	25.02 ± 0.00	78.50 ± 48.65	57.27 ± 21.75	-	2327.18 ± 3208.58	44.32 ± 0.00	57.69 ± 16.09	67.22 ± 27.33
SM3	1.00 ± 1.15	2.50 ± 2.12	3.50 ± 3.10	2.61 ± 2.63	-	1.50 ± 0.70	1.00 ± 0.00	2.88 ± 2.34	6.80 ± 6.18
	**Average Minutes/Day of PC Use during Lockdown**								
**Test**	**0**	**1–15**	**16–30**	**46–60**	**61–75**	**76–100**	**101–115**	**116–130**	**+146**
SM1.1	24.15 ± 5.06	21.05 ± 0.00	24.30 ± 6.11	22.15 ± 2.50	20.90 ± 0.00	20.64 ± 2.13	-	20.22 ± 3.50	22.03 ± 1.90
SM1.2	25.28 ± 4.15	22.35 ± 0.00	26.87 ± 7.11	26.52 ± 3.14	20.39 ± 0.00	24.41 ± 4.32	-	22.42 ± 3.38	25.30 ± 2.39
SM2	55.86 ± 30.59	34.25 ± 0.00	1203.40 ± 2261.86	64.53 ± 26.56	64.00 ± 0.00	58.45 ± 22.58	-	50.39 ± 12.67	73.53 ± 34.92
SM3	2.00 ± 2.35	3.00 ± 0.00	5.40 ± 5.03	2.44 ± 1.81	4.00 ± 0.00	5.00 ± 6.16	-	2.26 ± 2.31	3.75 ± 3.20
	**Average Minutes/Day of Tablet Use during Lockdown**								
**Test**	**0**	**1–15**	**16–30**	**46–60**	**61–75**	**76–100**	**101–115**	**116–130**	**+146**
SM1.1	21.53 ± 3.53	20.45 ± 0.84	23.28 ± 4.67	20.91 ± 3.16	-	21.5 ± 3.79	-	23.39 ± 5.45	20.09 ± 1.08
SM1.2	24.20 ± 3.96	22.95 ± 0.84	25.09 ± 6.13	24.04 ± 3.68	-	24.70 ± 1.83	-	24.30 ± 4.53	26.47 ± 2.66
SM2	406.87 ± 1258.82	38.44 ± 5.92	71.00 ± 37.68	68.05 ± 20.26	-	61.18 ± 3.98	-	48.62 ± 11.96	52.89 ± 34.03
SM3	21.53 ± 3.52	3.00 ± 0.00	3.50 ± 4.14	3.80 ± 2.58	-	1.00 ± 0.00	-	2.22 ± 1.56	2.25 ± 3.86
	**Average Minutes/Day of Mobile Phone Use during Lockdown**								
**Test**	**0**	**1–15**	**16–30**	**46–60**	**61–75**	**76–100**	**101–115**	**116–130**	**+146**
SM1.1	22.59 ± 4.64	20.08 ± 0.31	23.55 ± 2.82	22.12 ± 3.06	19.15 ± 0.00	-	-	21.92 ± 3.63	19.92 ± 2.88
SM1.2	24.42 ± 4.96	23.30 ± 0.34	22.46 ± 1.77	27.05 ± 2.95	28.32 ± 0.00	-	-	23.04 ± 3.64	25.76 ± 3.91
SM2	57.57 ± 24.78	72.81 ± 42.68	1574.86 ± 2616.39	65.14 ± 24.19	25.02 ± 0.00	-	-	57.55 ± 22.53	51.68 ± 23.03
SM3	2.76 ± 3.02	5.50 ± 3.53	1.33 ± 1.15	2.20 ± 1.30	1.00 ± 0.00	-	-	4.50 ± 4.93	2.50 ± 2.88

Note. Data is presented as mean ± standard deviation. SM1.1: skills manual 1.1—flipping pegs preferred hand; SM1.2: skill manual 1.2—flipping pegs non-preferred hand; SM2: skill manual 2—assembling a triangle; SM3: skill manual 3—drawing the layout.

## Data Availability

Data sharing not applicable.
